# Diversity of tomato leaf form provides novel insights into breeding

**DOI:** 10.1270/jsbbs.22061

**Published:** 2023-01-20

**Authors:** Hokuto Nakayama, Yasunori Ichihashi, Seisuke Kimura

**Affiliations:** 1 Graduate School of Science, Department of Biological Sciences, The University of Tokyo, Science Build. #2, 7-3-1 Hongo, Bunkyo-Ku, Tokyo 113-0033, Japan; 2 Department of Plant Biology, University of California Davis, One Shields Avenue, Davis, CA 95616, U.S.A.; 3 Riken BioResource Research Center, Tsukuba, Ibaraki 305-0074, Japan; 4 Faculty of Life Sciences, Kyoto Sangyo University, Kamigamo-motoyama, Kita-Ku, Kyoto 603-8555, Japan; 5 Center for Plant Sciences, Kyoto Sangyo University, Kamigamo-motoyama, Kita-Ku, Kyoto 603-8555, Japan

**Keywords:** breeding, evolution, evo-devo, leaf, leaf form diversification, tomato

## Abstract

Tomato (*Solanum lycopersicum* L.) is cultivated widely globally. The crop exhibits tremendous morphological variations because of its long breeding history. Apart from the commercial tomato varieties, wild species and heirlooms are grown in certain regions of the world. Since the fruit constitutes the edible part, much of the agronomical research is focused on it. However, recent studies have indicated that leaf morphology influences fruit quality. As leaves are specialized photosynthetic organs and the vascular systems transport the photosynthetic products to sink organs, the architectural characteristics of the leaves have a strong influence on the final fruit quality. Therefore, comprehensive research focusing on both the fruit and leaf morphology is required for further tomato breeding. This review summarizes an overview of knowledge of the basic tomato leaf development, morphological diversification, and molecular mechanisms behind them and emphasizes its importance in breeding. Finally, we discuss how these findings and knowledge can be applied to future tomato breeding.

## Introduction

Tomato (*Solanum lycopersicum* L.) is a vegetable crop cultivated worldwide (Bauchet and Causse 2012). Tomatoes are rich in sugars, vitamins, minerals, and other nutrients, including carotenoids, lycopene, and polyphenols (Vats *et al.* 2022). Tomato is a leading vegetable in the US; tomato consumption has substantially increased since the beginning of the last century (Razdan and Mattoo 2007). Apart from using fresh fruits of tomatoes in salads and soups, they are also used in processed foods such as tomato paste, sauce, powder, puree, juice, soups, etc. (Bergougnoux 2014, Razdan and Mattoo 2007). During the last two decades, tomato production has doubled (Bergougnoux 2014), with the recent global annual production of tomatoes reaching approximately 182.3 million tons (Vats *et al.* 2022), placing tomato as the second most important vegetable crop globally (Kulus 2022).

Since tomatoes have been bred for centuries, many quality attributes such as yield and fruit weight are strongly dependent on cultivars and growing conditions (Bergougnoux 2014, Causse *et al.* 2007). Consequently, numerous varieties and cultivars with different fruit shapes, sizes, colors, and flavors can now be found along with yield and fruit weight variations. These include strains called heirloom tomatoes, which are varieties passed down through several generations within a family or grown in a specific region (Goldman and Schrager 2008). Additionally, there are wild tomato species adapted to various environments, such as those especially found in arid regions (Moyle 2008). Approximately 75,000 varieties of tomatoes are cultivated and maintained in over 120 countries (Razdan and Mattoo 2007).

Moreover, tomato is a well-studied model plant, because of the wide range of morphological, physiological, and ecological varieties developed over centuries; the availability of plenty of classic mutants allows us to investigate individual traits. Additionally, the tomato has a relatively short reproductive cycle, is easy to cross and self-pollinate, and can be transformed with a high success rate (Arie *et al.* 2007, Quinet *et al.* 2019, Rothan *et al.* 2019). A tomato cultivar, Micro-Tom, which displays a very dwarf phenotype with small and red ripened fruits and can grow in chambers, is also available for use in molecular research (Martí *et al.* 2006, Okabe *et al.* 2011, Saito *et al.* 2011).

Whole-genome sequencing of the tomato is completed (The Tomato Genome Consortium 2012) with the data being updated regularly, providing a high-quality reference genome (https://solgenomics.net/organism/Solanum_lycopersicum/genome). Currently, there is a significant research focus on improving agronomical traits that are useful for crops such as plant growth, fruit quality, yield, and disease resistance (Soyk *et al.* 2017, Tieman *et al.* 2017, Thomazella *et al.* 2021). This has led to the introduction of a genome-edited tomato, developed using CRISPR-Cas9 (CRISPR associated protein 9) gene-editing technology, into the Japanese market (Waltz 2022). Additionally, gene-edited tomato plants that produce a precursor to vitamin D had opened up the possibility of providing an animal-free source of the crucial nutrient (Li *et al.* 2022). Thus, tomatoes are one of the crops where applied research is implemented in breeding for better varieties.

**Fig. 1.**
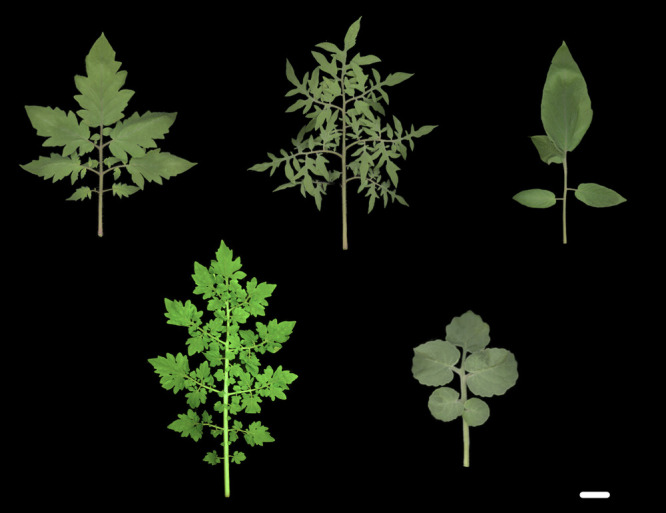
Leaf form variation in tomatoes. Upper; left to right. *Solanum lycopersicum* cv. M82; an heirloom tomato “Silvery Fir Tree”; an heirloom tomato “ABC potato leaf”. Lower; left to right *Solanum galapagense*; *Solanum pennellii*. Bar = 2 cm.

In particular, extensive studies were performed on tomato fruit phenotypes due to the crop nature (Akihiro *et al.* 2008, Frary *et al.* 2000, Fridman *et al.* 2004, Shinozaki *et al.* 2015). Interestingly, tomatoes are diverse not only in terms of their fruit but also in terms of their leaf morphology (Fig. 1); tomatoes are one of the crops that show morphological variations in cultivars/varieties. Recent studies have reported that leaflet shape and leaf-vein density affect fruit sugar content (BRIX) in tomatoes, indicating a trade-off between yield and quality; particularly, round/less lobed leaves have a positive effect on both fruit BRIX and yield (https://doi.org/10.1101/2021.03.01.433399, Rowland *et al.* 2020). Although the detailed mechanism that links leaf morphology to fruit quality is not fully understood—since leaves are photosynthetic organs and since vascular systems transport photosynthetic products—it is not hard to assume that these traits are related to the final fruit quality. These studies indicate that improving fruit quality requires not only research on the fruit itself but also that includes the relationship between leaf and fruit morphology. However, the importance of leaf-related traits has been overlooked in tomato breeding, indicating that the knowledge gained from fruit quality, leaf morphogenesis and its diversification should be used as an important component of breeding.

Here, we reviewed the existing knowledge on tomato leaf development, especially focusing on leaf form diversification resulting from changes in developmental processes at several different levels such as inter-species/-varieties. Finally, we discuss the potential and importance of employing phenotypes on leaf shape and architecture for future plant breeding programs of tomato.

## Basic tomato leaf development

**Fig. 2.**
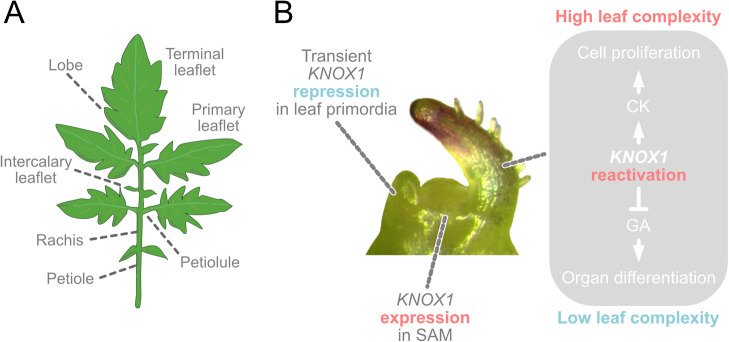
Basic compound leaf development. (A) Diagram of a compound leaf, indicating the leaf parts. (B) Schematic model of development focusing on *KNOX1* function in shoot apical meristem and leaf primordia.

Generally, leaves are classified into two distinct types: simple leaves and compound leaves: A simple leaf is a leaf with a single undivided blade; compound leaf is a leaf that comprises separate subunits such as leaflets that are separated by bladeless regions (e.g. petiole and petiolule; Fig. 2A) (Bar and Ori 2014, Efroni *et al.* 2010). Numerous studies using various model plants have elucidated the molecular mechanisms of leaf development (Maugarny-Calès and Laufs 2018, Nakayama *et al.* 2022a). Particularly, Arabidopsis (*Arabidopsis thaliana* (L.) Heynh.) and tomato have mainly contributed to the elucidation of developmental mechanisms for simple and compound leaves, respectively. These studies have revealed the several initial phases in leaf development: 1) leaf initiation, 2) establishment of adaxial-abaxial (ad-ab) polarity, 3) lamina initiation and outgrowth, and 4) cell enlargement. In recent years, it has also become evident that biomechanics such as cell elasticity, tensile stress direction, and geometries play an important role in leaf development (Nakayama *et al.* 2022b). Detailed molecular mechanisms of each of the developmental processes is an intense field of research and several reviews have been published (Bhatia *et al.* 2021, Conklin *et al.* 2019, Du *et al.* 2018, Maugarny-Calès and Laufs 2018, Nikolov *et al.* 2019, Tsukaya 2021).

The ancestral angiosperm is presumed to have simple leaves based on fossil records and previous phylogenetic studies (APG 2016, Bharathan *et al.* 2002, Hickey and Doyle 1977), and simple leaves are the most prevalent among angiosperm lineages (Geeta *et al.* 2012). Therefore, simple leaf development is considered the basic leaf development type in angiosperms. Previous studies have revealed that the basic developmental mechanisms of the compound leaf are quite similar to those of the simple leaf; in tomato, auxin transport is required for leaf initiation and proper phyllotaxis (Reinhardt *et al.* 2000); some homologs of genes involved in the establishment of ad-ab polarity in Arabidopsis are also involved in tomato (Wang *et al.* 2015). Recent studies reported an ortholog of *WUSCHEL-related homeobox1* (*WOX1*), which is involved in leaf lamina outgrowth in Arabidopsis, is involved in tomato leaflet outgrowth (Du *et al.* 2020, Nakayama *et al.* 2021, Wang *et al.* 2021, Zhang *et al.* 2020). However, certain mechanisms are unique to the compound leaf. One of the most important differences between the development of compound and simple leaves would be the repeated leaflet emergence. Previous studies have suggested that three processes regulate leaflet emergence: maintenance of the transient meristematic region, patterning of the new growth axis at the margin, and post-patterning differential growth of the new axis (Maugarny-Calès and Laufs 2018). In tomato, one of the factors involved in the maintenance of the transient meristematic region is the class I *KNOTTED-like homeobox* (*KNOX1*) genes. *KNOX1* plays an important role in the initiation and maintenance of the undifferentiated regions of shoot apical meristem (SAM) and is known to lose its expression from SAM during leaf initiation (Long *et al.* 1996). However, in some compound-leafed species, including tomato, *KNOX1* expression is reactivated in the developing leaf primordia (Fig. 2B) (Bharathan *et al.* 2002, Hareven *et al.* 1996). Indeed, *KNOX1* overexpression increased leaf complexity and marginal complexity in various plants (Chen *et al.* 1997, Hay and Tsiantis 2006). These facts indicate the role of *KNOX1* genes in compound leaf formation by establishing an indeterminate cell identity within developing primordia, which is thought to prolong the morphogenetic window and increase leaf complexity (Shani *et al.* 2009, Shwartz *et al.* 2016). In this context, tomato research has played an important role in elucidating the relationship between *KNOX1*, compound leaf development, and hormones. *Tomato KNOTTED1* (*Tkn1*) promotes cytokinin (CK) biosynthesis and represses the activity of gibberellins (GAs). Like other hormones, CK and GA have been reported to have various functions. Regarding leaf development, CK is important to promote cell proliferation, leading to prolonged morphogenesis and a delay in differentiation and senescence. The manipulation of CK levels led to a broad spectrum in leaf complexity in tomato (Shani *et al.* 2009, Shwartz *et al.* 2016). For instance, the overexpression of a CK biosynthesis gene *ISOPENTENYLTRANSFERASE 7* and a CK degradation gene *cytokinin oxidase* results in highly complex and reduced leaves, respectively (Shwartz *et al.* 2016). This is because CK is thought to promote the maintenance of prolonged morphogenetic activity at the tomato leaf margin. In contrast, GA shortens the morphogenetic window in leaf development by promoting cell differentiation. It is known that exogenous application of GA simplified leaf morphology; mutants showing increasing GA levels or GA response showed a reduction in leaf complexity in tomato (Shwartz *et al.* 2016). In *solanifolia* (*sf*) mutants, characteristic of leaves with low complexity and smooth margins, the application of a GA biosynthesis inhibitor increased leaf complexity, particularly at the leaf margin, indicating that increased GA level reduces complexity in leaf phenotype in *sf* (Sekhar and Sawhney 1990). A mutant of GA repressor protein in tomato, *procea*, exhibits reduced leaf complexity and smooth margins (Bassel *et al.* 2008, Jasinski *et al.* 2008, Shwartz *et al.* 2016). Additionally, CK and GA exhibit antagonistic activities in various developmental processes (Greenboim-Wainberg *et al.* 2005). This antagonistic relationship is known to be conserved in various plants (Greenboim-Wainberg *et al.* 2005, Weiss and Ori 2007).

Altogether, compound leaf development is influenced by hormones; from this perspective, the importance of *KNOX1* is notable as one of the factors that orchestrate multiple hormones. In the next section, we outline the role of *KNOX1* in the diversification of tomato leaf morphology.

## Genetic analysis revealed the basis of leaf form diversity among wild tomato species

The cultivated tomatoes we eat today are domesticated from wild tomato species. There are 13 wild tomato species, all of which are distributed around South America (Kimura and Sinha 2008, Rick 1991). Although the wild tomato species diverged from a common ancestor relatively recently (within 2.5 million years), their traits vary not only in terms of the size, shape, and color of fruits, flowers, and leaves but also in physiological characteristics such as disease resistance. Wild tomato species are distributed in various environments, including arid regions and coastal areas, and the diversity of the traits is thought to be the result of adaptation to such environments. The leaf morphology diversity—which can be observed in the number and size of lobes, the presence and depth of lobes, serrations on the leaf margins, leaf thickness, and leaf color—is extensive among wild tomato species. Investigating the genetic background of leaf shape differences among wild tomato species will provide important insights into the leaf morphology evolution.

Quantitative trait loci (QTL) analysis is an effective method for analyzing the genetic background of diversity of various traits (Lippman *et al.* 2007). Due to its agronomical importance, tomato has been subjected to QTL analysis for various traits. Because the edible part of tomato is a fruit, QTL analysis has been performed especially on fruit traits such as size, shape, yield, soluble solids content; some of the genes that determine the traits have been identified and their functions have been clarified (Frary *et al.* 2000, Fridman *et al.* 2004, Xiao *et al.* 2008). *Solanum pennellii* is the most distant infertile species of domesticated tomato; *S. pennellii* introgression Line (IL) populations, which consists of 76 ILs, were developed and widely used for QTL analysis (Eshed and Zamir 1994). The leaf shape of *S. pennllii* is simpler than that of cultivated tomatoes; the number of leaflets per leaf is fewer, and each leaflet has rounder and less serrated margin than cultivated tomatoes. QTL analysis was performed using the IL population to reveal the genetic basis of the differences in leaf morphology between *S. lycopersicum* and *S. pennellii*; 22 QTLs affecting leaf dissection were identified (Holtan and Hake 2003). The IL population was also used to analyze the quantitative basis of the evolution of leaf shape in wild tomatoes (Muir *et al.* 2014). Recently, a new backcross population (BILs) of *S. pennellii* and cultivated tomatoes were utilized for fine-mapping of QTLs involved in variations in leaf morphology (Fulop *et al.* 2016). These analyses identified several candidate genes involved in leaf shape variations, though the causal genes have not been identified.

An instance of the identification of genes responsible for the evolution of leaf morphology is the study of wild tomatoes endemic to the Galapagos Islands (Kimura *et al.* 2008). Two wild tomato species, *S. cheesmaniae*, and *S. galapagense* are distributed in the Galapagos Islands (Darwin *et al.* 2003). Despite the close relationship between the two species, significant differences in leaf morphology were observed. The leaf morphology of *S. cheesmaniae* is similar to that of cultivated tomatoes. In contrast, the leaf morphology of *S. galapagense* has a significantly greater number of leaflets with deeper lobes, resulting in an overall higher degree of leaf complexity. *S. galapagense* is distributed mainly in coastal regions of the islands that are salty with little water availability; the evolution of leaf shape is thought to be the result of adaptation to this harsh environment (Kimura *et al.* 2008). Map-based cloning revealed that the evolution in leaf morphology is caused by a deletion of 1 bp in the promoter of a gene called *PETROSELINUM* (*PTS*) (Kimura *et al.* 2008). PTS encodes a novel *KNOX1* gene that lacks a DNA-binding domain. Thus, *PTS* does not function as a transcription factor on its own though it can interact with BEL1-LIKE HOMEODOMAIN (BLH/BELL) proteins. In *S. galapagense*, *PTS* is ectopically expressed in young leaves due to the mutation in the promoter region and inhibits KNOX-BELL protein interaction, which is needed to reduce leaf complexity in the species. This is the first case in which the causes of diversity in leaf morphology among species have been identified at the gene level. It is also important as it reveals changes in gene regulatory networks due to *cis*-sequence variation, which contributed to the evolution of leaf morphology.

## Omics technology expands the feasibility of genetic control of leaf form in tomato

Genome sequencing followed by gene manipulation have helped understand the genetic regulatory mechanisms behind life processes in nature. Reverse genetics as a functional genomics approach provides powerful tools to analyze plant gene functions. Since plant developmental processes are underpinned by dynamic transcriptional regulation, reverse genetics approach in plant development usually starts with the transcriptome data, screening for genes with an interesting spatiotemporal expression pattern, and manipulation of the target genes to describe the biological function associated with the phenotypic trait (Ben-Amar *et al.* 2016). RNA-sequencing (RNA-seq) has provided a comprehensive understanding of transcriptome complexity with a broader range of expression levels and identification of novel transcripts (Wang *et al.* 2009). Improvements in RNA-seq library preparation methods such as the rapid, cost-effective protocol using breath capture for incorporating strand-specific adapters, for instance, have enabled us to obtain large-scale and high-resolution transcriptome data (Ichihashi *et al.* 2018, Townsley *et al.* 2015).

A decade ago, the genome of the inbred tomato cultivar was sequenced (The Tomato Genome Consortium 2012). Starting with this, a plethora of genomics information related to developmental and evolutionary contexts has been generated in tomato (Ichihashi and Sinha 2014, Ranjan *et al.* 2012). Especially, RNA-seq data of tomato genetic resources such as domesticated varieties, wild relatives, and introgression populations have been collected (Chitwood *et al.* 2013, Koenig *et al.* 2013, Liu *et al.* 2020, Ranjan *et al.* 2016), contributing to a holistic understanding of gene expression from the developmental to evolutionary time scale. RNA-seq provides catalogs of genes that function in tomato leaves, which can be used for reverse genetics. For example, global gene expression levels in leaves against different pathogen challenges such as that of viruses, fungi, bacteria, and oomycetes (Campos *et al.* 2021), as well as other environmental stress conditions such as shade, cold, and drought conditions (Chitwood *et al.* 2015, Zhou *et al.* 2019) were examined. The genes regulated under specific signal pathways such as cytokinin response and gene silencing mediated by microRNA were identified (Shi *et al.* 2013, Wang *et al.* 2015). Moreover, spatiotemporal expression patterns of tomato leaf development were characterized by time-series and laser capture microdissection analyses combined with transcriptome sequencing (Ichihashi *et al.* 2014, Martinez *et al.* 2021).

**Fig. 3.**
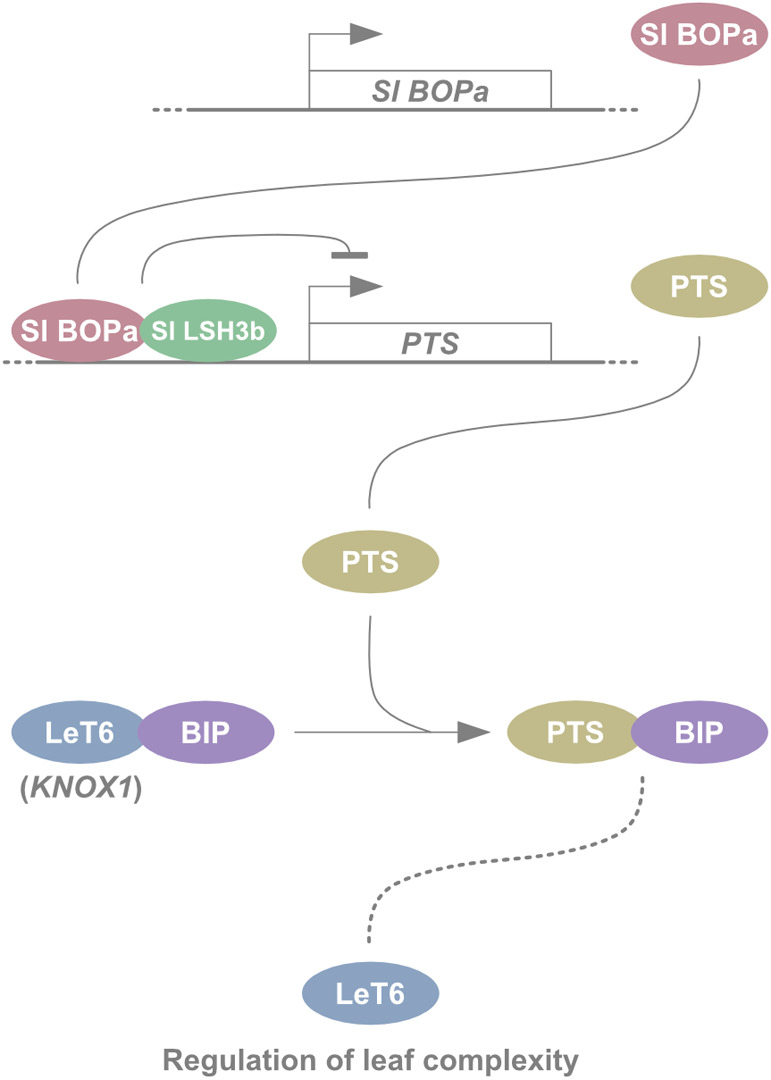
Schematic models of leaf developmental gene regulatory module leading to diversification in tomato at different levels. Sl BOPa interacts with Sl LSH3b and the complex directly regulates *PTS* expression. PTS regulates LeT6 at the protein level by competing with BIP. The released LeT6 regulates many genes involved in leaf development. Figure modified from Ichihashi *et al.* (2014).

In addition to the identification of key genes, gene co-expression patterns detected by transcriptome data can reveal the regulatory networks behind tomato leaf development (Nakayama *et al.* 2021). In a case study, correlation network analysis using spatiotemporal expression patterns of leaf development in tomato and two related wild species showed modules in the leaf developmental gene regulatory network (GCN) (Ichihashi *et al.* 2014). The resultant network showed the core network regulatory genes of global cell proliferation, while a peripheral gene network module showed *BLADE-ON-PETIOLE* (*BOP*) transcription factor that controls the core network by regulating the KNOTTED-like HOMEOBOX proteins through *PETROSELINUM* (*PTS*) and *LIGHT-DEPENDENT SHORT HYPOCOTYLS* (*LSH*) (Fig. 3) (Ichihashi *et al.* 2014). The expression levels of *BOP* transcripts were correlated with those of leaf complexities evident in *Solanum lycopersicum*, *S. pennellii*, and *S. habrochaites*. Allele-specific expression assays using F1 hybrids showed differences in *cis*-regulation of *BOP* between these species. Engineering of *BOP* expression in the three species reproduced the expected leaf complexity phenotypes associated with the *BOP* expression level (Ichihashi *et al.* 2014). Given that *BOP* suppresses the meristematic potential of the leaf margin at the early developmental window (Martinez *et al.* 2021), *BOP* acts as a genetic switch in leaflet initiation by regulating a broad range of leaf developmental genes. The direction of gene regulation under *BOP* gene network suggested that peripheral regions of the network, rather than core network hubs, might contribute to evolution of leaf morphology in tomato. In addition, the bottleneck location of the *KNOX1* gene in the network could explain the reason why *KNOX1* regulation was repeatedly co-opted to generate natural variations in leaf shape (Ichihashi and Tsukaya 2015). Thus, engineering leaf developmental regulatory networks using a reverse genetics approach could contribute to the discovery of cryptic traits in tomato breeding.

## Gene network-based analysis and genome comparison revealed the breeding process of an heirloom tomato

In addition to diversification of form among species, there is also diversification of form among varieties and cultivars in the case of crops. This is a result of domestication and subsequent improvement processes, which is characteristic of the adaptation of crops to agroecological niches and human preferences (Larson *et al.* 2014). Such morphological diversity among crops sometimes leads to new uses. For instance, *Brassica rapa* L. is an agronomically and economically important crop with various forms or “morphotypes”, such as leafy vegetables, turnips, and oilseed rape (McAlvay *et al.* 2021). This is an indication that understanding the process of morphological diversification within a crop is also important and helpful for breeding.

A recent study demonstrated that two *HOMEOBOX* genes are responsible for the leaf form diversification in an heirloom tomato, Silvery Fir Tree (SiFT) (Nakayama *et al.* 2021). SiFT is a traditional Russian heirloom tomato (Goldman and Schrager 2008) and shows a highly complex leaf phenotype, with narrower leaflets than those in processing tomatoes (e.g. M82; Fig. 1). To identify the genes responsible for the highly complex leaf phenotype, a cross between SiFT and M82 was generated as a mapping population; bulked segregant analysis (BSA) on an F2 population derived from a cross between M82 and SiFT was performed. Moreover, to narrow down the number of candidates for leaf morphology, a protein variation effect analyzer: PROVEAN (Choi *et al.* 2012), was used. The analyzer would help predict whether an amino acid substitution or an indel influenced the biological function of a protein. Combined BSA and PROVEAN analyses revealed that the SiFT genome has a deleterious variant in the *BIPINNATA* (*BIP*) (Solyc02g089940) gene, a BLH protein. BIP interacts with the KNOX1 LeT6 protein (Ezura *et al.* 2022), resulting in the localization of this heterodimer to the nucleus (Kimura *et al.* 2008). The 1-bp deletion at position 1674 within the homeobox domain generated a premature stop codon, and the BIP protein was truncated in SiFT. Interestingly, although the leaf complexity (LC) in *bip3* mutant is quite similar to that of SiFT, the two genotypes have distinct leaflet shapes. To confirm the difference, deep-learning-based nonlinear principal component analysis (PCA) with leaflet shapes in M82, bip3, and SiFT was conducted. The results indicated that the *bip3* leaflet shape was different from that of M82, however, it was not the same as SiFT (Nakayama *et al.* 2021).

Additionally, leaf vein density (LVD) in *bip3* was similar to that of M82 but differed from that of SiFT. These observations suggested that the mutation at the *BIP* locus was not sufficient to explain all leaf phenotypes seen in SiFT. To investigate the molecular basis for leaf phenotypes in SiFT, the GCN analysis based on RNA-seq data was performed. GCNs of M82 and SiFT RNA-seq data were compared and differential correlations between them were visualized with an R package DiffCorr (Fukushima 2013). DiffCorr analysis revealed a *WOX1* ortholog, *SOLANIFOLIA* (*SF*) (Solyc03g118770), which was significantly different between the M82 and SiFT GCNs (Nakayama *et al.* 2021). In Arabidopsis, *WOX1* and *PRESSED FLOWER* (*PRS*)/*WOX3* act in the middle domain where blade outgrowth and margin development occur after the establishment of adaxial/abaxial polarity (Nakata *et al.* 2012). *SF* was expressed at the margins of the leaf and leaflet primordia. qRT-PCR analysis revealed that the *SF* expression level in the SiFT leaf primordia was lower than M82 (Nakayama *et al.* 2021).

Additionally, morphological, and developmental analyses with *sf* mutant showed that *sf* is involved in leaf lamina expansion and leaf vascular development. Because SiFT has a *bip* mutation and *SF* repression, *bip* *sf* double mutant was generated where the double mutant exhibits highly complex, narrow leaves and low LVD. Therefore, a mutation at *bip* and *SF* repression created highly complex and narrower leaves with reduced leaf vein density in SiFT (Nakayama *et al.* 2021).

Since the genomes of various tomato accessions have been sequenced, the genome sequences can be used to unravel the history of breeding. Phylogenomic analysis with SiFT genome data suggested that the *bip* mutation in SiFT is likely a *de novo* mutation, instead of a standing genetic variation, and is unique in SiFT. Although SiFT produces edible fruits, SiFT is used as an ornamental and landscaping plant because of its unique leaf shape (Goldman and Schrager 2008); it is an unusual example of the use of tomatoes. The uniqueness of leaf shape in SiFT brought about by changes in two different *HOMEOBOX* genes likely led to new uses of this variety as an ornamental and landscaping plant. Therefore, in addition to the process of diversification of leaf morphology in tomatoes, the study with SiFT revealed the process of transition of the crop to other uses.

## Findings of leaf morphological diversity provide a new direction for tomato breeding

**Fig. 4.**
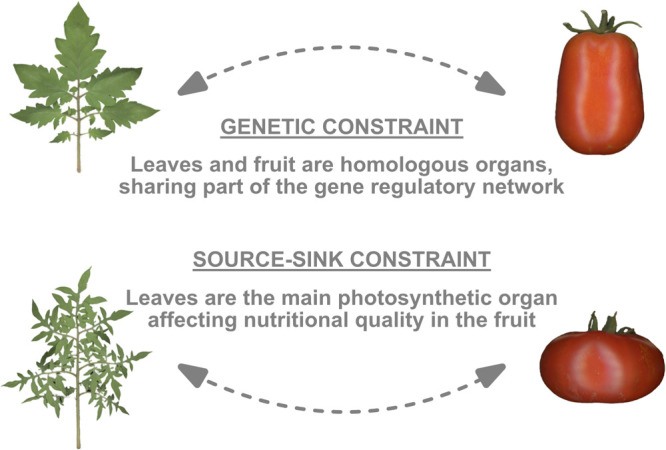
Relationships between leaf and fruit. There is a mutual relationship between leaf and fruit, with constraints as homologous organs and as a source-sink relationship.

Tomato is a major crop that exhibits wide morphological variations. Moreover, tomato is one of the major model plants. These characteristics have led to the elucidation of molecular mechanisms of compound leaf development and processes of leaf morphological diversification among species and varieties, as discussed above. However, the study of diversity in tomato leaf morphology could also be helpful for applications such as breeding. Because many studies have so far shown that leaf-related phenotypes including leaflet shape have direct or indirect effects on other organs, especially the fruits (Chitwood *et al.* 2014, Rowland *et al.* 2020). For instance, it is well known that defects in leaf morphology also affect fruit morphology. For instance, *SlBOP* RNAi mutants show a highly complex leaf phenotype as well as elongated fruit shape (Ichihashi *et al.* 2014). *sf* mutants show not only a narrow leaflet phenotype but a narrow and small fruit shape (Wang *et al.* 2021). This may be related to the fact that the floral organ is a homologous organ of the leaf (Weigel *et al.* 1992), indicating that leaves and fruit partially share a common genetic regulatory network (Fig. 4). In the case of tomatoes, the morphology of the carpel has a significant influence on the fruit shape (Kwon *et al.* 2022, Wang *et al.* 2021). Indeed, *SlBOP* RNAi and *sf* mutants show defects in carpel development (Ichihashi *et al.* 2014, Nakayama *et al.* 2021, Wang *et al.* 2021). It is known that fruit shape and size are important traits in fresh market tomatoes, influencing consumer preference and market value (Causse *et al.* 2010). Therefore, breeding for tomato fruit morphology should also be considered in relationship to leaf morphology, which may allow for further breeding of the tomato fruit morphology.

With respect to indirect effects of leaf morphology on fruit quality, studies with meta-analyses suggested that leaf shape, specifically round and less-lobed leaves, had a strong positive impact on BRIX (Chitwood *et al.* 2014, Rowland *et al.* 2020). Partial least squares path modeling revealed that leaf shape has a strong and direct correlation with the yield and BRIX (Rowland *et al.* 2020), indicating the importance of leaf shape to fruit quality in tomato. Interestingly, the analysis also revealed that photosynthesis negatively affects the yield while still positively contributing to fruit BRIX (Rowland *et al.* 2020). It is not hard to imagine that leaf morphology affects the efficiency of photosynthesis, however, since the mechanisms that regulate source-sink relations and sugar distribution are intricate and still not fully understood on a whole-plant physiological level (Osorio *et al.* 2014), future detailed studies are needed to determine why leaf morphology affects quality such as BRIX. However, a recent study involving grafting experiments to analyze the influence of leaf traits on fruit sugar level was performed which revealed that vein density was negatively correlated with fruit BRIX (https://doi.org/10.1101/2021.03.01.433399). In leaves, sugars synthesized through photosynthesis are loaded into leaf veins and then transported out of the leaves to the rest of the sink organs. Thus, leaf veins play an important role in the transportation of carbohydrates, and subsequent sugar accumulation in the fruits (Cataldo 1974, Haritatos *et al.* 2000). Several studies have shown that higher leaf vein density not only allows higher K^leaf^ (leaf permeability) and higher gas exchange rates per leaf area (Brodribb *et al.* 2010, Sack and Frole 2006) but also improves leaf loading (Russin and Evert 1984). Based on these studies, plants with higher leaf vein density should have higher sugar content in their fruits. However, this was not the case in the study reported by https://doi.org/10.1101/2021.03.01.433399, indicating that there are still many unanswered questions regarding the relationship between vascular bundle density and BRIX. Fortunately, leaf vein density and their architecture are correlated with hormones, especially auxin; relationship between auxin and leaf morphology has also been studied in tomato (Koenig *et al.* 2009, Shwartz *et al.* 2016). Thus, measurements of fruit quality using existing mutants for vascular and leaf morphology would be the first step which would be helpful in revealing the mechanisms underlying the relationship between leaf phenotypes and fruit quality, particularly in terms of BRIX.

Since the leaf is a photosynthetic organ and serves as a source organ that influences the sugar content of the fruit (Fig. 4), it is thought to have a morphology that optimizes its function under natural and artificial selections (Tsukaya 2018). On the other hand, fruit shape is also an essential parameter in the breeding process and a wide variety of forms can be found (Rodríguez *et al.* 2011). Therefore, the quality of many of the tomato varieties currently in the market are the result of the combined effects of leaf and fruit morphologies. As the diversity of leaf morphology and associated vascular architecture and the mechanisms of fruit quality control becomes clearer, it will be important to shift the attention towards those leaf phenotypes that have been overlooked in breeding experiments to improve tomato fruit quality.

To understand these mechanisms, technological advances are essential. As we discussed in this review, technological advances have been important in elucidating the mechanisms underlying the diversification of tomato leaf morphology. Especially, omics technologies such as genome, transcriptome, and metabolome analyses will become more important in the years ahead, because they can digitalize various biological information; the digital data would help understand the association between specific phenotypes and genetic information. Tomato has already been studied using many of these technologies and knowledge of leaf morphology and structure has been well-documented. Thus, the relationship between leaf morphology and fruit quality will be established using these advanced technologies, which would serve as one of the new indicators for improving tomato fruit quality. Beyond that, it is believed that breeding simulations coupled with physical-based models would be made available.

These will emphasize the unique characteristics of tomato as a crop, where basic research and breeding are closely intertwined and applied in practice and will contribute to an understanding of the importance of crop morphogenesis research in supporting the foundations of breeding science.

## Author Contribution Statement

All three authors contributed to writing and approved the final version of the manuscript. H.N. integrated all sections of the text and created the figures.
